# Preventive behaviors of respiratory infections in staff of hospital in Kazeroon, Fars, Iran: An application of protection motivation theory

**DOI:** 10.1111/crj.13791

**Published:** 2024-06-18

**Authors:** Tayebeh Rakhshani, Zohreh Shafiei, Samira Taravatmanesh, Seyyed Mansour Kashfi, Pooyan Afzali Harsini, Amirhossein Kamyab, Ali Khani Jeihooni

**Affiliations:** ^1^ Nutrition Research Center, Department of Public Health, School of Health Shiraz University of Medical Sciences Shiraz Iran; ^2^ Department of Public Health, School of Health Shiraz University of Medical Sciences Shiraz Iran; ^3^ Department of Public Health, School of Health Kermanshah University of Medical Sciences Kermanshah Iran; ^4^ Faculty of Medicine Fasa University of Medical Sciences Fasa Iran

**Keywords:** health behaviors, health education, Iran, psychological theory, respiratory infections

## Abstract

**Background:**

One of the most crucial and essential methods for the prevention and management of respiratory infections is for healthcare professionals to take precautions for their own safety. Using Protection Motivation Theory (PMT), the current study looked into effective elements influencing the staff at Kazeroon's Valiasr Hospital's preventive actions against respiratory diseases.

**Methods:**

One hundred ninety‐two male and 108 female employees of the Valiasr Hospital in Kazeroon, Iran, participated in this cross‐sectional study, in May 2022. Census data were used as the sample technique. A questionnaire based on the PMT and a questionnaire collecting demographic data served as the data collection method. The study's content validity was confirmed by 10 health education experts, and its reliability was assessed using internal consistency techniques, resulting in a Cronbach's alpha coefficient of 0.87.The statistical program SPSS 24 was used to examine the data using the independent *t* test, logistic regression, and Pearson correlation.

**Results:**

The average age was 34.11 ± 8.91 for men and 32.77 ± 6.09 for women. The majority of participants were married (73.3%), had university education (76.7%), and earned a monthly income between 10 and 15 million Tomans (75%). Notably, 97.7% of participants had received the COVID‐19 vaccine, and 77.7% had undergone training related to respiratory infections. The most common preventive practices included avoiding touching the eyes, noses, or mouths, wearing appropriate protective gear, and maintaining a safe distance of 1–2 m from others. Analysis of PMT constructs showed that participants had a generally positive perception toward preventive behaviors. Perceived vulnerability (*P* = 0.02), perceived cost (*P* = 0.03), and motivation (*P* = 0.001) were the three analyzed components that had the greatest impact on respiratory infection preventative behavior. Logistic regression revealed that perceived susceptibility, cost, and motivation significantly predicted the prevention of respiratory infections, with a predictive power of 45%. These findings highlight the importance of understanding the factors influencing preventive behaviors among hospital staff, from respiratory infections like COVID‐19.

**Conclusion:**

According to the findings, the personnel at Kazeroon's Valiasr Hospital wore gloves, goggles, and other appropriate personal protective equipment. The individuals' decision to wear personal protection equipment was also impacted by perceived susceptibility, cost, and motivation.

## Graphical abstract

This study used the protection motivation theory to analyze factors influencing healthcare professionals' preventive actions against respiratory diseases. 192 employees at Kazeroon's Valiasr Hospital in Iran were involved. Common preventive practices included avoiding contact, wearing protective gear, and maintaining a safe distance. Perceived vulnerability ability, perceived cost, and motivation significantly influenced respiratory infection preventative behaviors.

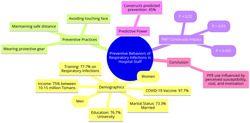

## 1 BACKGROUND

Respiratory infections are a significant public health concern globally, encompassing a diverse array of illnesses ranging from the common cold to severe respiratory diseases. Numerous illnesses fall under the category of these infections, such as tonsillitis, colds, COVID‐19, influenza, and lower respiratory tract infections.^1^, ^2^, ^3^ Fever, exhaustion, and a dry cough are typical signs of these illnesses. These are always followed by anorexia, muscle soreness, dyspnea, and other symptoms. Death may be due to progressive respiratory failure caused by alveolar damage.^4^ These infections have a high prevalence and can quickly infect many people, so governments must take several measures, such as emergency treatment of patients, quarantine of suspected and sick cases, protection of healthcare workers, and public health measures.^5^ According to the latest guidelines, these viruses could transfer through direct contact (through direct contact with respiratory droplets when a person coughs, sneezes, or talks), airborne transmission (through airborne particles known as aerosols), indirect contact (through contact with contaminated surfaces or objects), droplet nuclei, droplet spread, and through fecal‐oral route.^6^

One of the most dangerous respiratory infectious diseases that recently became a pandemic is COVID‐19. The pandemic was detected in Wuhan, China, in late 2019, being of the beta coronavirus genus. On January 11, 2019, the first death caused by the virus was reported in China. And in a short period of time, it spread to most countries in the world and led to more than 18 million infection cases and more than 700 000 deaths among health staff worldwide.^5^ Therefore, the need to prevent the diseases was raised, and the most important factor in controlling the transmission of the infection was preventive behaviors. These habits typically involve cleaning hands with soap and water or a disinfectant solution, not shaking hands, not kissing, cleaning bought goods, the interior of cars and homes, quarantining homes, and receiving vaccinations.^7^

Human resource management gurus have long stressed that human capital is one of the key pillars of every firm. Meanwhile, hospitals and other health facilities operate similarly to an industrial unit, using resources like capital, labor, technology, and management to produce a product known as the preservation, improvement, and return of human health; therefore, taking preventive measures by health staff for personal protection against the disease is one of the most important and necessary strategies in the prevention and control program of respiratory infections.^8^

Healthcare workers are in direct contact with patients infected with respiratory infections, so ensuring their safety is not only important in protecting them from being infected but also in preventing the spread of infection. To design preventive measures and control respiratory infections, effective factors influencing preventive behaviors against infections should be identified.^9^ The employment of preventive measures, such as education, knowledge‐building, and preventative skills for individual protection against the illness, is one of the most vital strategies for the prevention and control program of respiratory infections.^10^ Any health education program's planning process begins with selecting a model, and selecting an appropriate model ensures that the program is headed in the proper direction and stays on course.^11^

The protection motivation theory (PMT) is one of the instructional frameworks that have been suggested for health education. In this paradigm, it is claimed that accepting health activity (protective behavior) suggested against health risk is a direct action of the individual's goal to protect himself.^12^ Rogers postulated that fear influences five structures that determine protection motivation, or the intention to take protective action against a health threat, and that protection motivation ultimately results in health behavior. Perceived susceptibility (believing one is vulnerable to a health threat), perceived severity (thinking the threat is serious), perceived response efficacy (believing an adaptive response can eliminate the threat), perceived response costs (estimating the costs of engaging in the protected behavior, including money, people, time, and effort), and perceived self‐efficacy (thinking one can successfully carry out the protective behavior) are these five constructs.^12^

The PMT framework elucidates the cognitive processes underlying individuals' decision‐making regarding protective behaviors. Understanding these factors is essential for designing targeted interventions aimed at promoting protective behaviors among healthcare personnel, thereby enhancing infection control practices and mitigating the risk of respiratory infections within healthcare settings.^13^, ^14^, ^15^, ^16^ Furthermore, the application of this theory in analyzing the attitudes and beliefs of healthcare workers toward preventive behaviors holds significant promise for informing evidence‐based strategies to optimize infection prevention and control efforts in healthcare settings. Besides, the effectiveness of PMT has so far been proved in various studies regarding various health behaviors and prevention of diseases,^13^, ^14^, ^15^, ^16^ especially infectious and respiratory diseases such as COVID‐19. Given the established efficacy of PMT in elucidating various health behaviors and its relevance to infectious diseases like COVID‐19, employing this model in analyzing the beliefs and attitudes of healthcare workers toward preventive behaviors against respiratory infections provides a comprehensive approach to inform the design and implementation of targeted interventions aimed at promoting and sustaining these behaviors. Considering the increasing mortality and hospitalization due to respiratory infections among hospital staff, worry and anxiety of death, conflict between home and work environment, and distance of health staff from their family, and important role of the PMT in analysis of beliefs related to performing behaviors, this study was conducted to investigate effective factors on preventive behaviors by a group of hospital staff toward respiratory infections using the PMT.

## 2 METHODS

### 2.1 Study design and sampling

This cross‐sectional study was executed in Valiasr Hospital, Kazeroon City, Fars Province, Iran, in May 2022. Based on a similar study by Khazaei et al.,^17^ the use of the Cochran formula, taking into account the 20% dropout rate, with an alpha of 0.05 and a study power of 80%, was considered 300. The sampling method was a census. For this purpose, among the population were the employees of Valiasr Hospital; 300 employees were selected as available.

### 2.2 Inclusion and exclusion criteria

A minimum of 1 year of work experience, employment at the Valiasr Hospital in Kazeroon, and prior exposure to infectious diseases—particularly COVID‐19—were inclusion requirements for the study, whereas change of workplace and refusal to cooperate at any point throughout the study were considered as the exclusion criteria.

### 2.3 Ethical approval and consent to participate

The Human Research Ethics Committee at Shiraz University of Medical Sciences granted ethical permission with the ethical code: “IR.SUMS.SCHEANUT.REC.1401.049.” A written informed consent was acquired by each participant. Additionally, consent was received to digitally record every interview. Anonymity and confidentiality were guaranteed. Every method carried out in research projects involving human subjects complied with the 1964 Helsinki Declaration and the ethical guidelines established by the national and/or institutional research committee.

#### 2.3.1 Data collection tools

This section included three different questionnaires. This first questionnaire was the demographic information one. This questionnaire included age, sex, marital status, income, occupation, education, years of service, and use of personal protective equipment.

The second part included knowledge and respiratory infections preventive behavior questionnaires, which are describe below.

##### Knowledge

There were 15 questions in this part about knowledge of preventive measures against respiratory infections. The questions used a 5‐point Likert scale, with the options ranging from *strongly disagree* to *strongly disagree*.

##### Behavior

Nine questions about respiratory infection prevention were included in this part. The questions used a 5‐point Likert scale, with the options being *strongly disagree*, *disagree*, *agree*, and *no opinion*.

The third part measured the PMT constructs, which are explained in detail as follows:

Regarding the individuals' impression of the preventative activities of respiratory infections, *perceived sensitivity* was assessed using four questions based on a 5‐point Likert scale (*strongly agree* to *strongly disagree*).

A 5‐point Likert scale with seven options—*strongly agree*, *agree*, *disagree*, and *strongly disagree*—was used to assess the individuals' *perceived severity* of respiratory infection prevention activities.

Six questions about the individuals' perceptions of respiratory infection prevention behaviors—from *strongly agree* to *strongly disagree*—based on a 5‐point Likert scale were used to assess *perceived response cost*.

Five Likert‐type questions about the individuals' perceptions of the preventative behaviors against respiratory infections—*strongly agree* to *strongly disagree*—were used to assess *perceived reward*.

A 5‐point Likert scale with five possible answers—*strongly agree* to *strongly disagree*—was used to assess the individuals' *perceived self‐efficacy* in relation to their capacity to prevent respiratory infections.

Six questions on a 5‐point Likert scale (*strongly agree* to *strongly disagree*) were used to assess *perceived response cost*.

Three questions utilizing a 5‐point Likert scale (*strongly agree* to *strongly disagree*) were used to assess *fear*.

Six questions on a 5‐point Likert scale (*strongly agree* to *strongly disagree*) were used to assess *motivation*.

#### 2.3.2 Validity and reliability of the questionnaire

The study by Bashirian et al., whose validity and reliability were confirmed,^18^ served as the basis for the questionnaire. Ten experts in health education and promotion provided their comments to confirm the content validity of the current study, and internal consistency techniques were employed to assess the tool's reliability. Cronbach's alpha coefficient was calculated for the complete questionnaire using SPSS version 24 software. For perceived sensitivity, severity, self‐efficacy, reaction, cost, and behavior motivation, the corresponding Cronbach's alpha coefficients were 0.86, 0.88, 0.86, 0.85, 0.83, and 0.86. The total questionnaire coefficient was calculated to be 0.87.

#### 2.3.3 Data collection process

First, after obtaining the code of ethics from the ethics committee of Shiraz University of Medical Sciences and coordinating with the Valiasr Hospital of the city of Kazeroon and the principals of Valiasr Hospital, the researcher explained the goals of the project to the participants. Subsequently, the subjects filled out the surveys by themselves through the self‐report method.

#### 2.3.4 Data analysis

SPSS 24 was used to analyze the data. The Kolmogorov–Smirnov test was used to determine the data's normality first. Utilizing frequency, mean, and standard deviation, the data were described. The data were analyzed using Pearson correlation, independent *t* tests, and logistic regression analysis. A 0.05 significance threshold was used.

## 3 RESULTS

The subjects' demographic details are displayed in Table 1. The subjects' age was 33.69 ± 8.01 years with a standard deviation of 8.01 years, and their job experience was 9.29 ± 6.51 years. The participants' marital status was 73.3%; 54% of those with 76.7% of university education were in the medical area; 75% of the participants earned between 5 and 10 million Tomans per month. According to the results, 97.7% of participants had gotten the COVID‐19 vaccine, and 77.7% had received training regarding respiratory illnesses. Of those who stated they got their knowledge from the internet, 25% thought it was a reliable source to learn about respiratory illnesses (Table 1). Additionally, the results showed that the subjects' most common behaviors were avoiding touching their eyes, noses, or mouths, wearing work‐appropriate protective gear (masks, gloves, gans, and glasses), and, at 97.3% of the time, maintaining a safe distance of 1–2 m from coworkers, patients, and other visitors.

**TABLE 1:** Distribution of primary variables of study participants.

Variable		Number	Frequency (%)
Gender	Male	192	64
	Female	108	36
Marital status	Single	82	27.3
	Married	217	73.3
	Other	1	0.3
Education	High school	5	1.7
	College	65	21.7
	University	230	76.7
Related ward	Health staff	162	54
	Utility	43	14.3
	Administrative	35	11.7
	Laboratory	31	10.3
	Security	17	5.7
	Other	4	1.3
Monthly income	<5 million Tomans	8	2.7
	5–10 million Tomans	21	7
	10–15 million Tomans	225	75
	>15 million Tomans	53	17.7
Training related to respiratory infections	Yes	233	77.7
	No	67	22.3
Vaccination	Yes	293	97.7
	No	7	2.3
Being infected with respiratory infections	Yes	300	100
	No	0	0
Getting info on respiratory infections	Social networks	28	3/9
	Hoardings	34	11.3
	Pamphlets	46	15.3
	Internet	75	25
	Health staff	54	18
	Media	34	11.3
	Family	7	2.3
	Friends	20	6.7
	Others	2	0.7

Table 2 shows the mean and standard deviation of PMT constructs. Based on the table, all subjects had an above‐average mean score in the constructs (Table 2).

**TABLE 2:** Mean and standard deviation of PMT constructs.

Constructs	Number of questions	Mean	Standard deviation	Maximum–minimum
Perceived susceptibility	4	17.64	2.11	20–4
Perceived severity	7	26.72	4.79	35–7
Perceived response	7	28.90	3.46	35–7
Perceived reward	5	12.43	4.76	25–5
Perceived self‐efficacy	10	41.20	4.48	50–10
Perceived response cost	6	18.29	5.85	30–6
Fear	3	9.90	3.02	15–3
Motivation	6	24.92	3.48	30–6
Behavior	9	17.74	1.83	18–9
Knowledge	18	46.16	5.18	54–18

The association between the PMT components was ascertained through the use of Pearson's correlation test. According to Table 3, significant correlations were found between perceived susceptibility and all other PMT constructs. Perceived severity correlated significantly with susceptibility, response, reward, self‐efficacy, and fear. Additionally, all PMT constructs, except for self‐efficacy and knowledge, showed significant associations with perceived reward. Perceived self‐efficacy was significantly correlated with all constructs except perceived reward. Fear was notably correlated with several constructs, including susceptibility, severity, response, reward, self‐efficacy, and motivation. (Table 3).

**TABLE 3:** Correlation matrix between PMT constructs.

Constructs	Perceived susceptibility	Perceived severity	Perceived response	Perceived reward	Perceived self‐efficacy	Perceived response cost	Fear	Motivation	Behavior	Knowledge
Perceived susceptibility	1	0.20	0.29	−0.21	0.25	−0.12	−0.19	0.30	0.006	−0.20
Perceived severity	0.20	1	0.05	0.11	−0.05	−0.09	0.37	0.07	0.01	0.06
Perceived response	0.29	0.05	1	−0.12	0.56	0.07	0.22	0.56	0.08	0.13
Perceived reward	−0.21	0.11	−0.12	1	0.08	0.42	0.19	0.15	0.07	0.24
Perceived self‐efficacy	0.25	−0.05	0.56	−0.08	1	0.05	0.06	0.51	0.16	0.03
Perceived response cost	−0.12	0.09	0.07	0.42	0.05	1	0.07	0.04	0.1	−0.19
Fear	−0.19	0.37	0.22	0.19	−0.06	0.08	1	−0.18	0.08	0.26
Motivation	−0.30	0.07	0.56	−0.15	0.51	0.04	0.18	1	0.15	0.07
Behavior	0.006	0.01	0.08	0.07	0.16	0.1	0.08	0.15	1	0.15
Knowledge	0.20	0.06	0.13	0.24	0.03	0.19	0.26	0.07	0.15	1

*Note*: Pearson's correlation test (*P* < 0.05).

Logistic regression was used to determine the PMT construct with the highest predictive power for preventing respiratory infections. Logistic regression analysis revealed that perceived susceptibility (*P* = 0.02), perceived response cost (*P* = 0.03), and motivation (*P* = 0.001) significantly predicted preventive behaviors against respiratory infections. The PMT constructs collectively demonstrated a predictive power of 45% (Table 4).

**TABLE 4:** The results of the logistic regression to determine the predictive power of PMT constructs.

Components	Beta	Expected beta	Standard error	Degree of freedom	*P* value*
P.susceptibility	0.19	1.21	0.08	1	0.02
P.severity	−0.02	0.97	0.03	1	0.43
P.response	−0.06	0.94	0.06	1	0.31
P.reward	−0.02	0.97	0.04	1	0.54
P.self‐efficacy	0.05	1.05	0.04	1	0.18
P.response cost	−0.07	1.07	0.03	1	0.03
Fear	−0.07	0.93	0.05	1	0.20
Motivation	−0.19	0.82	0.05	1	0.001
Behavior	0.002	1.02	0.08	1	0.98
Knowledge	0.04	0.95	0.03	1	0.16

* Logistic regression (*P* < 0.05).

## 4 DISCUSSION

This study was conducted to assess the impact of PMT on the preventive behaviors of Valiasr Hospital staff in Kazeroon City, Iran, against respiratory infections. According to the findings, the professionals at the hospital donned gloves, goggles, and other proper personal protective equipment, indicating a conscientious approach to infection control. The decision of the participants to wear personal protective equipment was impacted by motivation, perceived cost, and perceived susceptibility. Comparing our results with existing literature, our findings align with previous studies emphasizing the significance of PMT constructs in predicting preventive behaviors. For instance, perceived susceptibility, severity, and self‐efficacy were identified as motivational factors for vaccine uptake, consistent with findings by Chen et al.^19^ Similarly, Prasetyo et al. highlighted the role of fear in increasing susceptibility, mirroring our observations regarding fear's correlation with PMT constructs.^20^

The results of the present investigation showed that the subjects' mean score on PMT components was higher than usual, which can be attributed to their successful completion of training courses, compliance with health measures, and questionnaire completion during the COVID‐19 pandemic. The subjects considered self‐efficacy as a part of their duties, and each of them was responsible for their own health and the large number of patients. Seeing the spread and occurrence of the disease every day, the fear of not observing personal protection was well observed in their behavior, and the necessary motivation was also created. Considering that all the websites were talking about respiratory infections, the subjects acquired full knowledge and showed appropriate behavior (use of personal protective equipment). Consistently, a study by Alhumaid et al.^21^ found a significant relationship between wearing personal protective equipment and understanding infection prevention. Regarding standard precautions, hand hygiene, and catheter care, it was determined that knowledge of infection control was sufficient. In a second study by Huang et al.,^22^ perceived knowledge was strongly connected with coping appraisal, and coping appraisal was significantly correlated with adaptive reaction.

Regarding information sources, our findings suggest a reliance on the internet among healthcare workers for respiratory infection‐related knowledge, which could be attributed to its accessibility and timely updates, especially during the COVID‐19 pandemic. Considering that these people are familiar with various types of respiratory infections, they may refer to valid medical and scientific internet sources and update their information. Likewise, during the COVID‐19 pandemic, many domestic and foreign sites upload their information daily, so it is natural that subjects also declare the internet as their most important source of information. This finding contrasts with Girma et al.'s observations on discrepancies in knowledge and application of preventive measures, highlighting variations in information‐seeking behavior across different contexts and populations.^23^

The findings showed that there was a strong correlation between all constructs and perceived vulnerability, and perceived severity demonstrated a substantial link with perceived sensitivity, responsiveness, reward, self‐efficacy, and fear. Additionally, there was a strong correlation between the perceived response and each of the model's constructs. These findings aligned with those of research conducted by Okuhara et al.,^24^ Yeganeh et al.,^25^ and Emami et al.^26^ To justify this finding, we should state: Based on the results, the most frequent behaviors by the subjects included avoiding touching the face (eyes, nose, and mouth), using protective equipment suitable for the work environment (mask, gloves, gans, and glasses), and keeping a distance of 1–2 m with colleagues, patients, and other visitors in unnecessary cases, with a frequency of 97.3%. In a study by Chen et al., perceived susceptibility, perceived severity, and self‐efficacy were among the factors motivating people to receive the vaccine, which is a means of personal protection against respiratory infections.^19^ This is consistent with the results of our study. In another study by Prasetyo et al., the fear of infection had a significant effect on increasing susceptibility.^20^

Notably, the correlation between perceived reward and all constructs except self‐efficacy and knowledge underscores the multifaceted nature of perceived rewards in shaping behavior. However, these differed from the findings of research by Azadeh et al.,^27^ which may have been caused by differences in the statistical population, questionnaire design, and sample size. This problem may have its roots in people's strong desire to avoid doing things that make them feel exposed; the same is true of contagious diseases. In this sense, an individual who recognizes their vulnerability attempts to avert the illness by adhering to a set of health guidelines. Moreover, there was a strong correlation between perceived reward, perceived vulnerability, and perceived cost. Besides, perceived sensitivity, perceived severity, perceived response, perceived reward, self‐efficacy, and motivation were all significantly correlated with fear. The strong correlation between the constructs of the PMT was also proved by findings of a study conducted by Khazaei et al.^17^

Furthermore, our study revealed a significant association between perceived vulnerability and preventive behaviors. Additionally, knowledge was significantly correlated with every construct of the PMT except perceived severity and self‐efficacy. Most of the participants have passed recent training courses on the prevention of respiratory infections. Moreover, the subjects are educated in the field of health; it is natural that they have the necessary knowledge and skills to prevent these diseases. Additionally, due to their work environment, where they are constantly in contact with all kinds of patients and infections, they are required to comply with health principles. In line with our results, the studies by Kim et al.,^28^ Ochie et al.,^29^ and Tu et al.^30^ also highlight the major contribution of knowledge between knowledge and the constructs of the PMT.

Also, perceived cost had a significant relationship with preventive behavior, which is consistent with the results of studies that was conducted with the aim of assessing the constructs of the PMT in preventive behaviors of respiratory infections and especially COVID‐19, by Grano et al.,^31^ Navabi et al.,^32^ Martin‐Lapoirie et al.,^33^ and Calcagni et al.^34^ Due to the fact that the subjects had passed all the training courses to prevent respiratory infections, all the participants were also infected with some kind of respiratory infection. Given the work environment, the way respiratory infections are transmitted is completely normal; every day, subjects face a large number of patients suffering from respiratory infections who are easily infected.

In the present study, motivation had a substantial relationship with preventive behavior, which is consistent with the results of studies by Yoon et al.,^35^ Acar et al.,^36^ Meng et al.,^37^ and Leung et al.^38^ Finally, in terms of predictive power, the results of logistic regression reported a 45% predictive ability of PMT constructs, which was consistent with Rezaeipandari et al.'s findings.^39^ On the other hand, the results obtained from Khishkar's study reported the predictive power of PMT toward respiratory infections to be 37%, which does not align with our results.^40^ The reason behind this disparity could be due to variations in study populations.

## 5 STRENGTHS AND WEAKNESSES

The use of a PMT questionnaire, the participation of the medical personnel in the study, and the researcher's attendance during the entire data collection procedure are among the study's strengths. Because this framework is widely used in health behavior research, it allows researchers to understand and predict individuals' responses to health threats and their adoption of preventive behaviors.

Moreover, the PMT questionnaire covers various constructs, and this comprehensive assessment enables a thorough examination of the factors influencing preventive behaviors against respiratory infections. Apart from its validity and reliability, the use of the PMT allowed us compare our results with other studies that have employed similar measures. Finally, by identifying specific factors such as perceived susceptibility, severity, and self‐efficacy, interventions can be tailored to address the unique needs and concerns of the target population, thereby increasing the effectiveness of public health initiatives.

On the other hand, the inability to extrapolate the findings to other research populations is one of the study's shortcomings.

## 6 CONCLUSION

Our findings underscore the commendable adherence of hospital staff to infection control measures, such as consistent use of personal protective equipment. Furthermore, the study illuminates the pivotal role of PMT constructs in shaping preventive behaviors. Specifically, perceived susceptibility, perceived cost, and motivation emerged as significant determinants influencing the utilization of personal protective equipment among hospital staff. This highlights the importance of addressing individuals' perceptions of vulnerability and the perceived barriers to adopting preventive measures, while simultaneously fostering motivation to uphold recommended behaviors.

Our findings also emphasize the impact of training and education on knowledge acquisition and behavioral outcomes. This underscores the importance of ongoing education initiatives aimed at equipping healthcare workers with the necessary knowledge and skills to effectively combat respiratory infections. Additionally, the reliance on internet sources for information highlights the need for accessible and credible resources to support healthcare workers in staying updated on best practices and emerging threats in infection control.

The study highlights the importance of training and education in shaping individuals' knowledge and behaviors related to respiratory infection prevention. In addition, because motivation and perceived susceptibility were found to have a significant relationship with preventive behaviors, future interventions aimed at enhancing motivation and personal susceptibility seem necessary. The reliance on the internet as a primary source of information underscores reliable sources of information and provide healthcare workers with easy access to credible resources for respiratory infection prevention. Further research could delve deeper into the nuances of PMT constructs and their impact on preventive behaviors in different contexts and populations.

## AUTHOR CONTRIBUTIONS

Tayebeh Rakhshani, Zohreh Shafiei, Samira Taravatmanesh, Seyyed Mansour Kashfi, Pooyan Afzali Harsini, Amirhossein Kamyab, and Ali Khani Jeihooni managed data collection, carried out data analysis, and wrote the text in addition to helping with study conception and design. In addition to helping with data analysis and paper review, Ali Khani Jeihooni and Tayebeh Rakhshani planned and designed the project. Tayebeh Rakhshani, Zohreh Shafiei, Samira Taravatmanesh, Seyyed Mansour Kashfi, Pooyan Afzali Harsini, Amirhossein Kamyab, and Ali Khani Jeihooni reviewed the paper and helped with research conceptualization. Every writer has read and approved the final manuscript.

## CONFLICT OF INTEREST STATEMENT

Not applicable.

## CONSENT FOR PUBLICATION

Not applicable.

## ETHICS STATEMENT

The Human Research Ethics Committee at Shiraz University of Medical Sciences granted ethical permission with the ethical code: “IR.SUMS.SCHEANUT.REC.1401.049.” A written informed consent was acquired by each participant. Additionally, consent was received to digitally record every interview. Anonymity and confidentiality were guaranteed. Every method carried out in research projects involving human subjects complied with the 1964 Helsinki Declaration and the ethical guidelines established by the national and/or institutional research committee.

## Data Availability

The data used would be available from the corresponding author on request.
